# Cycling in synchrony

**DOI:** 10.7554/eLife.52884

**Published:** 2019-12-02

**Authors:** Míriam Osés-Ruiz, Nicholas J Talbot

**Affiliations:** The Sainsbury LaboratoryUniversity of East AngliaNorwichUnited Kingdom

**Keywords:** pheromone signalling, Ustilago maydis, cell cycle, virulence, fungal development, Plant disease, Other

## Abstract

The corn smut fungus uses two different mechanisms to control its cell cycle when it is infecting plants.

**Related research article** Bardetti P, Castanheira SM, Valerius O, Braus GH, Pérez-Martín J. 2019. Cytoplasmic retention and degradation of a mitotic inducer enable plant infection by a pathogenic fungus. *eLife* **8**:e48943. doi: 10.7554/eLife.48943

Plant diseases are a major threat to global food security (Savary et al., 2019), but we need to know more about these diseases if we want to stop them spreading. Current disease control strategies are not very effective, because of the ability of microbes (such as bacteria or fungi) to develop resistance to anti-microbial drugs and overcome plant immunity.

Many disease-causing fungi undergo highly complex life cycles, with some involving more than one host plant species, as well as sexual and asexual stages of reproduction (Li et al., 2019). A fascinating example of this complexity is *Ustilago maydis*, the fungus that causes the disease called corn smut. For *U. maydis* to infect corn plants, two haploid cells belonging to different mating types must fuse together on the surface of a leaf to form an infectious cell known as an appressorium. This cell can then rupture the surface and invade the plant’s tissue. The end result is deformed corn cobs, covered in black sooty spores – if you have dined on the delicacy *Huitlacoche* in a Mexican restaurant, then you’ll have eaten them! However, if you are a corn grower, a field of deformed cobs is a disaster.

The behavior of *U. maydis* on a leaf surface is quite remarkable: although infection relies on two cells fusing, the nuclei inside these cells do not fuse with each other, so the new cell contains two nuclei. This means that the cell must somehow control and synchronize two cell cycles to ensure its survival and development. Now, in eLife, José Pérez-Martín and co-workers at the Institute of Functional and Genomic Biology in Salamanca and Georg August University in Göttingen – including Paola Bardetti as first author – report how this amazing feat is accomplished (Bardetti et al., 2019).

In eukaryotes the cell cycle goes through four main stages to produce two identical daughter cells: G1, S (when DNA replication occurs), G2, and M (mitosis; Figure 1A). Progression through these stages depends on the activity of a family of proteins called cyclin-dependent kinases (CDKs) in association with partner proteins called cyclins. Mechanisms controlling the activity of CDK1 form the basis of cell cycle regulation (Nurse, 2012): the kinase Wee1 stops the cell cycle by phosphorylating CDK1 and blocking its activity, and the phosphatase Cdc25 allows the cycle to start again by dephosphorylating CDK1 (Figure 1A).

**Figure 1.**
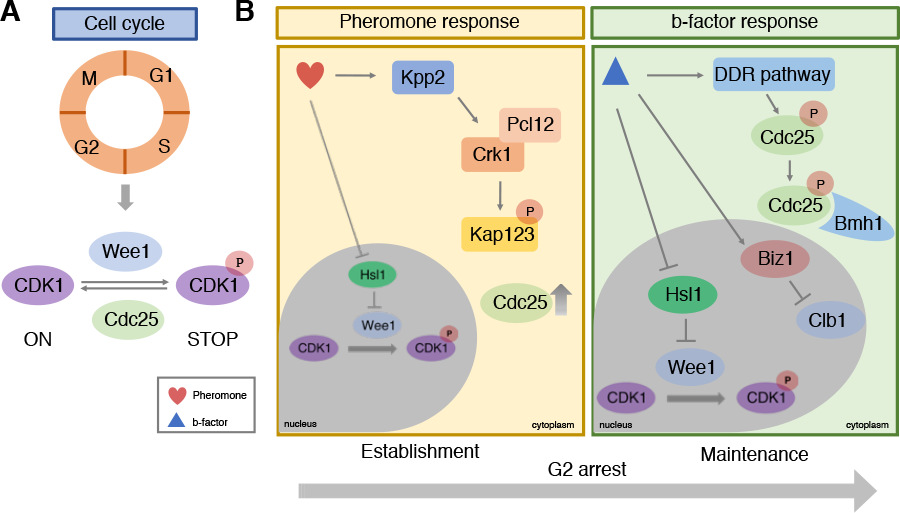
Cell cycle control during infection by the corn smut fungus *Ustilago maydis.* (**A**) Progression through the four stages of the eukaryotic cell cycle depends on the activity of cyclin-dependent kinases (CDKs): when CDK1 is phosphorylated by Wee1 it becomes inactive and stops the cell cycle from progressing. De-phosphorylation by the Cdc25 phosphatase re-activates CDK1, allowing the cell cycle to continue to the next stage. (**B**) During infection, *U. maydis* arrests its cell cycle at the G2 stage using two distinct mechanisms. In the first mechanism (left) pheromones released from mating cells prior to fusion activate the Kpp2 signaling cascade, causing the Crk1-Pcl12 complex to phosphorylate the importin protein Kap123 so it can no longer import Cdc25 into the nucleus. Activation of Kpp2 also triggers down-regulation of the *hls1* gene, which encodes a kinase that normally represses Wee1. This leads to increased Wee1 activity and inhibition of CDK1. Once the mating cells have fused, a second mechanism regulated by a homeodomain transcriptional regulator called b-factor (right) keeps the cell cycle arrested at G2 by: i) activating the DNA damage response (DDR) which causes Cdc25 to become phosphorylated and bind to a protein called Bmh1: this interaction ensures that Cdc25 is retained in the cytoplasm and cannot activate CDK1; ii) triggering the transcription of the *biz1* gene, which in turn represses the transcription of the protein Clb1, which is needed to activate CDK1; iii) repressing the transcription of *hsl1* gene that leads to an increase in phosphorylated inactive CDK1.

Initial work in model yeast revealed that when cells fuse during mating, synchronization of the cell cycle normally occurs by arresting the cell cycle at the G1 stage (Chang and Herskowitz, 1990). In *U. maydis*, however, the cell cycle is stopped at the G2 stage after DNA replication has occurred. Bardetti et al. showed that cell cycle arrest at G2 is triggered by two distinct mechanisms. The first is employed before fusion and involves pheromones released from the two mating cells. The second occurs only after the cells have fused and is necessary to maintain synchrony of the two nuclei that now occupy the same cell.

Previous work has shown that pheromone perception triggers G2 cell cycle arrest by activating the Kpp2 signaling cascade (Müller et al., 2003). Bardetti et al. revealed that Kpp2 inhibits a protein known as Kap123 from importing Cdc25 into the cell nucleus (Figure 1B). This causes Cdc25 levels in the nucleus to decrease, leading to higher levels of inactive CDK1, thus causing the cell cycle to stop. Activated Kpp2 also increases the levels of inactive phosphorylated CDK1 by repressing the transcription of the *hls1* gene that normally blocks the activity of Wee1.

At this point, as cells of opposite mating type move towards each other, the Cdc25 proteins within their cytoplasm begin to degrade as a result of Kpp2-dependent phosphorylation. This ensures that the cell cycle remains stopped. Once the two cells have fused together, they begin to develop branching filaments that can invade the plant tissue. The formation of these filaments relies on a homeodomain transcriptional regulator called b-factor, which keeps the cell cycle arrested at the G2 stage (Urban et al., 1996). Bardetti et al. showed that this sustained G2 cell cycle arrest involves three separate layers of control on CDK1 activity (Figure 1B). The first retains Cdc25 in the cytoplasm by binding it to a cytoplasmic protein called Bmh1, so it cannot activate CDK1 in the nucleus; the second represses the transcription of *hsl1*, leading to an increase in Wee1 activity; and the third turns on a gene called *biz1* which blocks the transcription of a protein known as Clb1 that activates CDK1.

It is likely that *U. maydis* employs two different arrest mechanisms to ensure that cell cycle control can be rapidly reversed before the cells fuse, but then maintained after fusion as the filaments develop. For example, if the two mating cells on the leaf surface fail to meet each other they will likely need to return to their vegetative state (perhaps to try and mate again!). This could easily be achieved by reversing the phosphorylation of importin proteins, such as Kapp123, which would lead to an increase of Cdc25 in the nucleus. By contrast, once a filament is formed, both mating partners are fully committed to each other, and the three layers of control used to sustain G2 arrest therefore need to be more difficult to reverse. Consistent with this idea, Bardetti et al. showed that the ability of *U. maydis* to infect a corn plant depended on the b-factor mechanism and not the pheromone mechanism.

This work highlights how important cell cycle regulation is for controlling the development process used by fungi to infect plants. Recent reports suggest that other infectious structures involved in some of the world’s most devastating crop diseases also rely on cell cycle check-points during their formation (Osés-Ruiz et al., 2017; Fukada and Kubo, 2015). Learning more about the methods used to control the cell cycle in these systems will be essential for identifying new ways to control these diseases.
